# Risk Factors for Depression in Empty Nesters: A Cross-Sectional Study in a Coastal City of Zhejiang Province and China

**DOI:** 10.3390/ijerph16214106

**Published:** 2019-10-24

**Authors:** Yecheng Yao, Gangqiang Ding, Liaoliao Wang, Ye Jin, Jianwei Lin, Yujia Zhai, Tao Zhang, Fan He, Weigang Fan

**Affiliations:** 1 National Institute for Nutrition and Health, Chinese Center for Disease Control and Prevention, Beijing 100050, China; yaoyc@ninh.chinacdc.cn (Y.Y.); dinggq@chinacdc.cn (G.D.); 2 Wenling Center for Disease Control and Prevention, Wenling 317500, China; wangliaoliao333@163.com (L.W.); qq61997374@163.com (Y.J.); wlcdcwsw@163.com (J.L.); 3 Zhejiang Provincial Center for Disease Control and Prevention, Hangzhou 310051, China; yjzhai@cdc.zj.cn (Y.Z.); tzhang@cdc.zj.cn (T.Z.); fhe@cdc.zj.cn (F.H.)

**Keywords:** risk factors, depression, empty nesters

## Abstract

The elderly are susceptible to depression, especially empty nesters. This study investigated the risk factors associated with depression in empty nesters. The participants were selected via multi-stage random cluster sampling. Depression symptoms were assessed using the Geriatric Depression Scale. The questionnaire surveyed demographic characteristics, underlying diseases, behaviors and lifestyles, negative life experiences, social support, self-care ability, etc. Chronic health conditions, such as being overweight/obese, smoking cessation, cancer, and chronic pain, as well as negative life experiences, such as the death of a loved one and financial loss, increased the risk of depression in empty nesters. In addition, the incapability of performing self-care also increased the risk of depression in empty nesters. The prevalence of depression in empty nesters was high. Being overweight/obese, cancer, chronic pain, smoking cessation, the death of a loved one, financial loss, and the incapability to deliver self-care were risk factors for depression in empty nesters. It is recommended that comprehensive measures be taken to enhance health interventions for this population, including encouraging a proper diet and physical activity for weight control, providing psychological counseling after negative life experiences, and teaching correct methods of smoking cessation.

## 1. Introduction

The aging population phenomenon is a major social problem around the globe in the 21st century. At present, most people can expect to live to 60 or older as the youth mortality rate is greatly reduced and life expectancy continues to grow [1]. Combined with the decline in the fertility rate, the increase in life expectancy is leading to a rapid aging of the world’s population [1]. In China, as of the end of 2018, the number of people aged 60 or older was close to 250 million, accounting for 17.9% of the total population [2]. In 2015, the “Family Development Report of China (2015)” issued by the National Health and Family Planning Commission showed that empty nesters accounted for half of all elderly people. It is estimated that the proportion of empty-nest families will account for 90% of elderly families in 2030, when the elderly families in China will be empty-nested across the board [3,4].

On a global scale, the burden of depression continues to increase [5]. Depression is a common mental disorder, and approximately 300 million people of all ages suffer from it worldwide. Depression is the leading cause of disability in the world and it is a major factor contributing to the global burden of disease [6]. Depression can lead to suicide in the worst cases [7]. Depression is expected to be the main cause of disease burden in 2030 [4]. The elderly are more susceptible to depression than other populations, especially empty nesters [8]. In China, the prevalence of depression in the elderly is worrying. In 2012, the results of an investigation using the Geriatric Mental State examination among 4265 community elderly people conducted in four provinces in China showed that the detection rate of depression symptoms in empty nesters was 8.18%, which was higher than the 6.31% for non-empty nesters [9]. Empty nesters are more likely to develop depression than average elderly people [10]. The depression problem of empty nesters has become a major social problem related to the health status of China’s aging population [4].

Empty nesters are susceptible to different adverse conditions and they are confronted with age-related problems, such as chronic diseases [5,11,12,13,14] and irreversible reductions in cognitive function [15]. In addition, more and more studies have shown that social support [16], behaviors and lifestyles [17,18], self-care ability [19,20], negative life experiences [14,18,21,22], obesity [23], etc., are the main risk factors for depression in empty nesters. Some studies have also shown that gender, age, occupation, education level, marital status, etc. affect the occurrence of depression [7,24,25,26,27,28,29]. Therefore, this study aimed to assess the prevalence and distribution of depression in empty nesters in a coastal city of China through cross-sectional questionnaires and physical examinations, and to explore the risk factors associated with depression in empty nesters in order to provide an effective theoretical basis for resolving the problem.

## 2. Materials and Methods

### 2.1. Participants

This study defined empty nesters as individuals living alone for more than six months a year due to their children having left home (for work, study, marriage, etc.), being childless, being widow/divorced, etc., including empty-nested couples living together and empty-nested individuals living by themselves. The exclusion criteria were as follows: (1) Individuals who had lost relatives and friends within two months before the investigation; (2) individuals who had taken psychotropic drugs or received treatment for mental illnesses within two months before the investigation; (3) individuals who had significant physical illnesses and could not participate in the survey; and (4) individuals who were not willing to participate in this research.

The sample size was calculated using the following formula:

$$n=\frac{DE\times N\times p\times (1-p)}{\frac{d^{2}}{Z_{1-\frac{\alpha }{2}}^{2}}\times (N-1)+p\times (1-p)}$$

DE stood for “design effect” and was assigned as 1.5, N was the total number of empty nesters (50,000), the expected prevalence rate p was assigned as 50%, d was assigned as 5%, and α = 0.05. The calculated sample size was 572. Using the multi-stage random sampling method, 8 townships/subdistricts (6 townships and 2 subdistricts) in the city were randomly selected, 1 village/residential committee was randomly selected from each township/subdistrict, and 66 empty nesters were selected as a research unit from each village/residential committee. Empty nesters aged 60 or older were then selected as research participants, with the total sample size being 813 people.

A one-to-one and face-to-face survey was conducted for each eligible empty nester using a self-designed questionnaire. All participants signed an informed consent form, and this research was approved by the Bioethical Committee at Taizhou Tumor Hospital (SL2019007), Zhejiang Province, PR China.

### 2.2. Assessment of Depression

The Geriatric Depression Scale (GDS) compiled by Brink et al. in 1982 and standardized for the elderly [30] was used, and it includes 30 items with a total score of 30 points. Specifically, 0 to 10 points indicates no depression, 11 to 20 points indicates mild depression, and 21 to 30 points indicates moderate to severe depression. The validated Chinese version of the GDS was used in this study.

### 2.3. Investigation of Related Factors

Related factors used in this study were divided into six groups: Demographic characteristics, underlying diseases, behaviors and lifestyles, negative life experiences, social support evaluation, and self-care ability assessment.

#### 2.3.1. Demographic Characteristics

Gender, age (divided into three groups: 60 to 69 years old, 70 to 79 years old, and over 80 years old), occupation (cultivation, housework, and other occupations), education level (illiterate, primary school, and junior high school, and above), marital status (married, widow/divorced, and other), etc. were described in the demographic characteristics section.

#### 2.3.2. Underlying Diseases

Underlying diseases included investigation of hypertension, diabetes, dyslipidemia, coronary heart disease, stroke, chronic respiratory diseases, cancer, chronic pain (more than three months’ duration with daily or almost daily pain), and physical discomfort (participants were asked whether there had been any physical discomfort in the last two weeks).

#### 2.3.3. Behaviors and Lifestyles

Smoking status (never, still smoking, or smoking cessation), drinking status (never, still drinking, or drinking cessation), exercise (whether the participant exercised in their spare time), and use of micronutrients (whether the participant had regularly taken oral vitamins, calcium tablets, or other trace element supplements in the last year) were included in the behaviors and lifestyles section.

#### 2.3.4. Negative Life Experiences

Whether the empty nesters had experienced health changes, economic difficulties, the death of a loved one, loss of property, sad experiences, conflicts between relatives or friends, or terror in the last two years was asked in the negative life experiences section.

#### 2.3.5. Social Support Evaluation

The Social Support Rating Scale has 10 items, covering objective support (3 items), subjective support (4 items), and support utilization (3 items), with a total score of 40 points. Higher scores indicated high social support. It is generally considered that a total score of less than 20 points indicates limited social support, 20 to 30 points indicates general social support, and 30 to 40 points indicates satisfactory social support.

#### 2.3.6. Self-Care Ability Assessment

The Barthel index rating scale was used to evaluate the participants’ self-care ability. The scale includes 10 items with a total score of 100 points. A score of no more than 40 points indicates heavy dependence, where the participant relies totally on the care of others; a score of 41 to 60 points indicates moderate dependence, where the participant relies mostly on the care of others; a score of 61 to 99 points indicates light dependence, where the participant relies partly on the care of others; and a score of 100 points indicates no dependence, where the participant does not need others to take care of them. The validated Chinese version was used in this study.

### 2.4. Statistical Analysis

The percentages of depression in populations with different characteristics were estimated based on the complex sampling method, and a correlation analysis was performed using the Rao–Scott chi-square test. The OR (odds ratio) value of each factor associated with depressive symptoms was calculated by univariate weighted logistic regression analysis, and the confounding effect of various factors was further controlled by multivariate weighted logistic regression analysis. Statistical tests (two-tailed, α = 0.05) and all analyses were performed using the SPSS 20.0 software (SPSS Inc., Chicago, IL, USA).

## 3. Results

A total of 813 people (326 males and 487 females) were enrolled in the study. The GDS was used to evaluate the depressive symptoms of empty nesters. The estimated prevalence of depressive symptoms in empty nesters was 46.5% (95% CI: 8.6–54.6%). The prevalence of depressive symptoms in different populations is shown in Table 1. The prevalence of depressive symptoms in men was lower than the prevalence of depressive symptoms in women, and the weighted prevalence rates were 41.2% (95% CI: 33.0–49.9%) and 50.0% (95% CI: 41.8–58.2%), respectively. The weighted prevalence of depressive symptoms in overweight/obese participants was 55.8% (95% CI: 46.1–59.9%), which was higher than weighted prevalence of depressive symptoms in participants with lean/normal weight. The weighted prevalence of depressive symptoms in participants engaged in housework was 56.8% (95% CI: 45.6–67.2%), which was significantly higher than individuals with cultivation or other occupations. The weighted prevalence of depressive symptoms among religious believers (49.7%, 95% CI: 40.9–58.5%) was significantly higher than weighted prevalence of depressive symptoms among non-religious believers (29.0%, 95% CI: 17.3–44.4%). The prevalence of depression in participants with chronic diseases, such as dyslipidemia (62.0%, 95% CI: 50.2–72.5%), chronic respiratory diseases (54.1%, 95% CI: 50.3–57.8%), and cancer (84.5%, 95% CI: 55.0–96.1%), was high. Smokers (39.4%, 95% CI: 27.0–53.2%) and drinkers (33.5%, 95% CI: 21.7–47.9%) showed a lower prevalence of depressive symptoms than non-smokers/drinkers or participants who had quit smoking/drinking. In terms of negative life experiences, individuals who had experienced the death of a loved one (73.7%, 95% CI: 60.4–83.8%), financial losses (80.0%, 95% CI: 53.5–93.3%), and sad experiences (72.9%, 95% CI: 55.9–85.1%) demonstrated a high prevalence of depressive symptoms.

The results of the multivariate weighted logistic regression analysis of depressive symptoms (Table 2) showed that being overweight/obese could increase the risk of depressive symptoms (adjusted OR = 1.64, 95% CI: 1.04–2.56) compared to people with a normal BMI. Compared to participants with cultivation, individuals engaged in housework demonstrated a higher risk of depressive symptoms (adjusted OR = 2.48, 95% CI: 1.86–3.31). Religious people showed a higher risk of depressive symptoms than individuals without religious beliefs (adjusted OR = 2.24, 95% CI: 1.19–4.20). At the same time, chronic diseases, such as cancer (adjusted OR = 5.19, 95% CI: 1.14–23.56) and chronic pain (adjusted OR = 1.60, 95% CI: 1.18–2.17), could significantly increase the risk of depressive symptoms. Compared to non-smokers, quitters were prone to depressive symptoms (adjusted OR = 3.08, 95% CI: 1.89–5.02); drinking appeared to produce a certain protective effect against depressive symptoms (adjusted OR = 0.60, 95% CI: 0.37–0.99). Negative life experiences, such as the death of a loved one (adjusted OR = 2.26, 95% CI: 1.75–2.91) and financial losses (adjusted OR = 5.30, 95% CI: 2.99–9.39), could significantly increase the risk of depressive symptoms in empty nesters. In addition, the incapability of providing self-care (adjusted OR = 1.53, 95% CI: 1.10–2.13) also increased the risk of depressive symptoms in empty nesters.

## 4. Discussion

This study showed that the prevalence of depressive symptoms in empty nesters reached 47%, which was significantly higher than the rate of 23.6% seen in the general elderly population. This finding was consistent with the findings of other studies [10,11,25], which may be due to the fact that, compared to non-empty nesters, empty nesters report lower incomes, a higher prevalence of chronic diseases, and no intimate siblings, friends, or even neighbors, which may increase their susceptibility to discomfort and anxiety and a higher risk of depression.

This study showed that the occurrence of depression in empty nesters was related to chronic conditions, such as being overweight/obese, religious beliefs, cancer, and chronic pain, as well as practices such as smoking and drinking. Being overweight/obese is a common problem among the elderly in China. In 2012, the rate of overweight residents aged 60 or older in China was 31.9% and the obesity rate was 11.6%, which were 7.6% and 2.7% higher than in 2002, respectively [31]. Being overweight/obese is a risk factor for many chronic diseases, which, to some extent, increases the psychological burden of the elderly. Chronic diseases damage the health of empty nesters and they also reduce their quality of life [32]. Empty nesters are likely to complain about emptiness and helplessness in life and feel that they lack friends and, therefore, are likely to be depressed [33,34]. Because of the poor prognosis and high mortality rate of many cancers, people generally have negative thoughts toward cancer. Therefore, after the diagnosis of cancer, the elderly, including empty nesters, are prone to anxiety and depression [19]. A strong association was found between cancer and the risk of depressive symptoms. Similarly, chronic pain is a long-term affliction in the elderly that negatively impacts their normal life, sleep, rest, and even psychology, making them prone to depressive symptoms [23,35]. Seemingly, empty nesters with religious beliefs are at a high risk of developing depressive symptoms, which may be related to the search for mental support by empty nesters after the occurrence of diseases or negative life experiences. In other words, many empty nesters develop religious beliefs after experiencing depressive symptoms or corresponding risk factors [36,37]. Smoking and drinking can relieve the psychological stress and anxiety of the elderly to a certain extent. On the other hand, however, smoking cessation may cause certain depressive symptoms in the elderly [38,39], and this may be due to the fact that, for some elderly, smoking was one of their pleasures that has now been taken away from them.

This study showed that the negative life experiences of losing loved ones and financial losses are risk factors for depression in empty nesters. These experiences often negatively impact the mental state of the elderly, making them prone to anger, loneliness, sadness, etc. Moreover, empty nesters cannot share their negative life events with other individuals due to the lack of an effective social support system, and negative coping strategies are often used, which can easily lead to symptoms of depression [1,40]. Chan et al. [41] found that negative life events, as stressors, were positively correlated with depression and they were potential risk factors for depression in the elderly. One study [42] showed that the number of negative life events was directly proportional to the risk of developing senile depression, and the number of negative events had an additive effect on senile depression. Therefore, negative life events, such as the death of a loved one and financial losses (e.g., due to natural disasters such as earthquakes), are likely to induce depressive symptoms in empty nesters [43,44].

High economic levels tend to lead to better health conditions and high life satisfaction [45,46]. A study on health and life satisfaction among the elderly showed that their perceptions of life satisfaction varied according to their health status [47], while life satisfaction was negatively correlated with depression [48]. Economic hardship may limit the ability of empty nesters to live independently and meet their social needs or desires [49]. Studies have shown that, compared with non-empty nesters, the income of empty nesters is lower, which may prevent them from interacting with friends or family in life or work, limiting their opportunities to increase their satisfaction with these aspects, increasing the incidence of depression [45].

The incapability of providing self-care is a risk factor for depression in elderly people. Self-care ability is the basis for the elderly to maintain social activities. Elderly people with impaired self-care ability will reduce their activities due to the weakening of the body, leading to a decline in physical flexibility and social adaptability. Consequently, elderly people will experience declines in their confidence to care for themselves, leading to fear and depression [50]. Self-care ability is closely related to the occurrence of depression [51]. The risk of depression in empty nesters with impaired self-care ability is significantly higher than the risk of depression in elderly people with normal self-care ability, which may be due to impaired self-care ability and leaves empty nesters dependent on their family and society. If these individuals are not properly cared for, they will experience negative emotions, such as helplessness or worthlessness, leading to depressive symptoms [52]. Therefore, improving the self-care ability of empty nesters is of great significance to the prevention and treatment of depression in this population.

## 5. Conclusions

This study investigated the prevalence of depressive symptoms and the corresponding risk factors in empty nesters and provided primary evidence for exploring the health problems of this population and proposing interventions. The main limitation of this study is that it was a cross-sectional investigation, which cannot determine the chronological order of factors and depressive symptoms necessary to determine causality. Through the study, it was found that the prevalence of depression in empty nesters was high and its occurrence was related to many factors. Being overweight/obese, cancer, chronic pain, smoking cessation, the death of a loved one, financial losses, and the incapability of providing self-care are risk factors for depression in empty nesters. We suggested that comprehensive measures should be taken to enhance health interventions for empty nesters, which include encouraging proper diets and physical activity to help them lose weight, delivering psychological counseling for negative life experiences, and teaching correct methods of smoking cessation. Further in-depth research on the major health issues of empty nesters is also suggested [42,53], such as lower limb atherosclerosis disease (LEAD), which is associated with increased risk of incident heart failure and may cause subsequent heart failure [54].

## Figures and Tables

**Table 1:** General characteristics of included empty-nested older adults.

Characteristics	Depressive Symptoms				Rao–Scott Chi-square	*p*-Value
	n	%	Weighted %	95% CI of Weighted %		
**Sex**						
**Male**	157	48.2	41.2	33.0–49.9	14.501	0.007
Female	263	54.0	50.0	41.8–58.2		
**Age Group (years)**						
60–69	140	49.5	48.7	39.5–57.9	1.366	0.288
70–79	183	54.3	47.0	37.0–57.1		
≥80	97	50.3	42.8	36.4–49.4		
**Body Mass Index (BMI, kg/m^2^)**						
Normal 18.5–24.9	187	48.1	42.1	32.8–52.1	12.876	0.003
Lean <18.5	19	44.2	37.6	34.5–40.8		
Overweight/obese ≥ 25.0	203	55.8	53.0	46.1–59.9		
**Educational Level**						
Illiterate	261	54.6	48.6	40.2–57.0	3.963	0.064
Primary	133	48.5	45.5	35.2–56.1		
Secondary and higher	26	42.6	35.8	28.1–44.3		
**Occupation**						
Cultivation	120	45.6	37.0	31.2–43.3	17.099	0.003
Housework	239	61.3	56.8	45.6–67.2		
Other	61	38.1	34.5	23.6–47.2		
**Marital Status**						
Married	259	48.9	44.7	38.1–51.4	3.486	0.069
Widow/Divorced	148	57.1	50.3	39.0–61.5		
Other	13	54.2	49.3	35.3–63.4		
**Religious Belief**						
Yes	365	54.0	49.7	40.9–58.5	10.987	0.013
No	55	40.1	29.0	17.3–44.4		
**Hypertension**						
Yes	186	52.5	47.1	38.4–56.0	0.390	0.552
No	234	51.0	46.1	38.4–54.0		
**Diabetes Mellitus**						
Yes	62	58.5	54.3	43.8–64.5	5.517	0.051
No	358	50.6	45.5	37.5–53.7		
**Dyslipidemia**						
**Yes**	41	64.1	62.0	50.2–72.5	31.993	0.001
No	379	50.6	45.2	37.6–53.0		
**Coronary Heart Disease**						
Yes	38	58.5	53.3	34.6–71.1	1.640	0.241
No	382	51.1	46.0	38.7–53.4		
**Stroke**						
Yes	24	63.2	59.5	36.5–79.1	2.885	0.133
No	396	51.1	45.9	38.4–53.5		
**Chronic Respiratory Disease**						
Yes	27	56.3	54.1	50.3–57.8	11.553	0.011
No	393	51.4	45.9	37.8–54.2		
**Cancer**						
Yes	8	66.7	84.5	55.0–96.1	10.392	0.015
No	412	51.4	46.0	38.1–54.2		
**Chronic Pain**						
Yes	126	61.8	56.3	41.1–70.4	5.249	0.056
No	294	48.3	42.7	35.8–49.8		
**Discomfort**						
Yes	67	53.2	46.3	30.8–62.6	0.002	0.966
No	353	51.4	46.6	39.4–53.9		
**Smoking Status**						
Never	337	51.5	46.7	39.4–54.2	6.437	0.017
Current	55	48.7	39.4	27.0–53.2		
Former	28	62.2	59.0	48.6–68.7		
**Drinking Status**						
Never	348	53.8	48.7	40.7–56.7	6.411	0.012
Current	48	39.3	33.5	21.7–47.9		
Former	24	54.5	52.0	39.7–64.1		
**Physical Activity**						
Yes	115	51.1	46.1	34.2–58.5	0.023	0.884
No	305	51.9	46.7	39.4–54.2		
**Nutrient Supplements**						
Yes	55	55.0	47.0	33.4–61.1	0.016	0.904
No	365	51.2	46.5	39.0–54.2		
**Worry About Children**						
Yes	180	63.8	54.2	47.4–60.9	4.568	0.070
No	240	45.2	42.8	32.0–54.3		
**Change of Health Status**						
Yes	157	65.7	51.0	37.8–64.0	0.772	0.409
No	263	45.8	44.8	35.3–54.7		
**Financial Hardship**						
Yes	85	63.4	48.2	34.9–61.7	0.115	0.745
No	335	49.3	46.2	37.9–54.8		
**Death of a Close Relative**						
Yes	58	77.3	73.7	60.4–83.8	39.344	<0.001
No	362	49.1	43.9	36.9–51.2		
**Unexpected Financial Loss**						
Yes	33	86.8	80.0	53.5–93.3	13.298	0.008
No	387	49.9	45.3	37.7–53.3		
**Sad Experiences**						
Yes	56	80.0	72.9	55.9–85.1	20.092	0.003
No	364	49.0	44.6	37.0–52.5		
**Conflicts between Relatives or Friends**						
Yes	22	81.5	65.2	28.3–89.9	1.657	0.239
No	398	50.6	46.1	38.2–54.2		
**Experience of Terror**						
Yes	24	64.9	42.5	20.6–67.8	0.183	0.682
No	396	51.0	46.8	39.2–54.5		
**SSRS Score**						
<20	29	48.3	44.6	31.8–58.1	1.483	0.263
20–29	178	59.9	53.1	46.8–59.3		
≥20	213	47.1	43.4	30.8–57.0		
**Self-Care Ability**						
Yes	396	51.4	46.3	38.2–54.7	1.630	0.242
No	24	61.5	53.4	44.9–61.6		

**Table 2:** Multifactor weighted logistic regression analysis of depressive symptoms.

Risk Factors	OR (95% CI)	*p*-Value	Adjusted OR (95% CI)	*p*-Value
**Sex**				
Male	0.70 (0.56–0.87)	0.007	1.17 (0.98–1.40)	0.072
**Age Group (years)**				
60–69				
70–79	0.93 (0.68–1.27)	0.620	0.94 (0.70–1.28)	0.668
≥80	0.79 (0.53–1.17)	0.197	0.78 (0.47–1.28)	0.277
**Body Mass Index (BMI, kg/m^2^)**				
Normal				
Lean	0.83 (0.59–1.15)	0.220	1.00 (0.73–1.37)	0.992
Overweight/obese	1.55 (1.16–2.07)	0.009	1.64 (1.04–2.56)	0.032
**Educational Level**				
Illiterate				
Primary	0.88 (0.71–1.09)	0.210	0.82 (0.66–1.01)	0.057
Secondary and higher	0.59 (0.37–0.94)	0.032	0.62 (0.34–1.15)	0.110
**Occupation**				
Cultivation				
Housework	2.23 (1.35–3.69)	0.007	2.48 (1.86–3.31)	<0.001
Other	0.90 (0.49–1.62)	0.674	0.89 (0.6–1.32)	0.508
**Marital Status**				
Married				
Widow/Divorced	1.25 (1.00–1.57)	0.051	0.87 (0.53–1.44)	0.545
Other	1.20 (0.72–2.01)	0.422	1.28 (0.39–4.23)	0.634
**Religious Belief**				
Yes	2.42 (1.27–4.62)	0.014	2.24 (1.19–4.20)	0.019
**Hypertension**				
Yes	1.04 (0.89–1.22)	0.552	0.83 (0.65–1.06)	0.117
**Diabetes Mellitus**				
Yes	1.43 (1.00–2.04)	0.052	1.24 (0.81–1.91)	0.277
**Dyslipidemia**				
Yes	1.98 (1.48–2.64)	0.001	1.45 (0.88–2.40)	0.125
**Coronary Heart Disease**				
Yes	1.34 (0.78–2.32)	0.242	1.11 (0.68–1.82)	0.624
**Stroke**				
Yes	1.74 (0.8–3.79)	0.137	1.70 (0.50–5.72)	0.339
**Chronic Respiratory Disease**				
Yes	1.39 (1.10–1.75)	0.012	1.14 (0.88–1.48)	0.272
**Cancer**				
Yes	6.4 (1.35–30.44)	0.026	5.19 (1.14–23.56)	0.037
**Chronic Pain**				
Yes	1.73 (0.98–3.06)	0.057	1.60 (1.18–2.17)	0.008
**Discomfort**				
Yes	0.99 (0.56–1.74)	0.966	0.75 (0.44–1.27)	0.235
**Smoking Status**				
Never				
Current	0.74 (0.52–1.06)	0.087	1.09 (0.61–1.97)	0.732
Former	1.64 (1.12–2.41)	0.018	3.08 (1.89–5.02)	0.001
**Drinking Status**				
Never				
Current	0.53 (0.34–0.83)	0.012	0.60 (0.37–0.99)	0.045
Former	1.14 (0.60–2.19)	0.639	0.74 (0.32–1.68)	0.409
**Physical Activity**				
Yes	0.98 (0.67–1.42)	0.884	1.02 (0.80–1.30)	0.854
**Nutrient Supplements**				
Yes	1.02 (0.68–1.53)	0.904	0.75 (0.55–1.01)	0.055
**Worry About Children**				
Yes	1.58 (0.95–2.64)	0.071	1.88 (0.84–4.25)	0.108
**Change of Health Status**				
Yes	1.28 (0.66–2.49)	0.409	0.92 (0.52–1.63)	0.753
**Financial Hardship**				
Yes	1.08 (0.62–1.90)	0.745	0.77 (0.49–1.22)	0.220
**Death of a Close Relative**				
Yes	3.59 (2.16–5.95)	0.001	2.26 (1.75–2.91)	<0.001
**Unexpected Financial Loss**				
Yes	4.81 (1.57–14.71)	0.013	5.30 (2.99–9.39)	<0.001
**Sad Experiences**				
Yes	3.33 (1.71–6.49)	0.004	0.90 (0.61–1.35)	0.563
**Conflicts between Relatives or Friends**				
Yes	2.19 (0.50–9.60)	0.249	0.96 (0.55–1.67)	0.869
**Experience of Terror**				
Yes	0.84 (0.32–2.20)	0.682	0.41 (0.26–0.64)	0.002
**SSRS Score**				
<20				
20–29	1.41 (0.94–2.10)	0.082	1.52 (0.89–2.61)	0.108
≥20	0.96 (0.36–2.56)	0.916	0.74 (0.27–2.01)	0.499
**Self-Care Ability**				
No	1.32 (0.79–2.23)	0.244	1.53 (1.10–2.13)	0.019
